# Imaging Features of Patients with Hepatocellular Carcinoma and Portal Vein Tumor Thrombosis Surviving Beyond 1 Year After Combined Therapy

**DOI:** 10.3390/diagnostics16010115

**Published:** 2025-12-31

**Authors:** Wei-Ming Lin, Hui-Ling Huang, Sheng-Nan Lu, Tse-Yen Yang, Hao-Chung Wang, Sheng-Lung Hsu, Chia-Hsuan Lai, Te-Sheng Chang

**Affiliations:** 1 Department of Diagnostic Radiology, Chang Gung Memorial Hospital, Chiayi 613016, Taiwan; weiming276@gmail.com (W.-M.L.);; 2 Graduate Institute of Clinical Medical Sciences, College of Medicine, Chang Gung University, Taoyuan 333323, Taiwan; 3 Department of Nursing, Chang Gung Memorial Hospital, Chiayi 613016, Taiwan; 4 Department of Hepato-Gastroenterology, Division of Internal Medicine, Kaohsiung Chang Gung Memorial Hospital, Kaohsiung 833253, Taiwan; 5 Department of Medical Research, China Medical University Hospital, Taichung 404327, Taiwan; 6 Department of Radiation Oncology, Chang Gung Memorial Hospital, Chiayi 613016, Taiwan; 8802041@cgmh.org.tw; 7 Department of Hepatology and Gastroenterology, Division of Internal Medicine, Chang Gung Memorial Hospital, Chiayi 613016, Taiwan; 8 College of Medicine, Chang Gung University, Taoyuan 333323, Taiwan; 9 School of Medicine, College of Medicine, National Sun Yat-sen University, Kaohsiung 80424, Taiwan

**Keywords:** hepatocellular carcinoma (HCC), portal vein tumor thrombus (PVTT), computed tomography (CT), magnetic resonance imaging (MRI), radiation therapy (RT), response evaluation criteria in solid tumors (RECIST) version 1.1

## Abstract

**Background/Objectives:** Portal vein tumor thrombus (PVTT) is a severe complication of hepatocellular carcinoma (HCC) and is associated with poor outcomes. This study aimed to describe the imaging and clinical characteristics observed among HCC patients with PVTT who survived longer than one year following combined systemic therapy and radiotherapy. **Methods:** This retrospective, single-center study included 26 consecutive HCC patients with PVTT who survived more than one year after combined treatment. Baseline characteristics included PVTT extent classified according to the Liver Cancer Study Group of Japan—VP1 (segmental portal vein invasion), VP2 (second-order portal vein invasion), VP3 (first-order portal vein invasion), and VP4 (main portal trunk or contralateral PV invasion) and liver function assessed by Child–Pugh class and ALBI grade. Contrast-enhanced CT or MRI was evaluated at baseline and 6 months after treatment using RECIST 1.1 criteria. **Results:** The cohort was predominantly male (69%), and most patients had extensive PVTT (VP3–VP4, *n* = 19). Preserved liver function was common at baseline (Child–Pugh class A, *n* = 24; ALBI grade I, *n* = 14). Tumor response was observed in 23 patients (88%) during follow-up. Frequently observed post-treatment imaging findings included portal vein recanalization (*n* = 12), collateral circulation (present in 7 patients at baseline and 6 at follow-up), and compensatory liver hypertrophy (*n* = 6). **Conclusions:** Among HCC patients with PVTT who survived longer than one year after combined therapy, portal vein recanalization, collateral circulation, and compensatory liver hypertrophy were commonly observed imaging features. Given the retrospective design and survivor-selection nature of the study, these findings should be interpreted as descriptive observations rather than evidence of treatment efficacy or prognostic determinants.

## 1. Introduction

Hepatocellular carcinoma (HCC) is a primary liver malignancy. At initial diagnosis, 10–40% of patients with HCC present with a portal vein tumor thrombus (PVTT) [1]. The occurrence of PVTT is associated with high morbidity and mortality rates, as evidenced by a median survival rate of 2–5 months despite optimal supportive treatment [2]. Therefore, effective therapies must be explored given the variability in prognostic factors for this condition [3]. Although many treatment options are available, no universally accepted guidelines currently exist for patients with HCC and PVTT [4,5,6]. Such guidelines should consider the differences in clinical practices, resource availability, and patient characteristics. Despite these efforts, consensus on the best treatment approach remains elusive, consequently rendering the management of HCC with PVTT a remarkable clinical challenge.

The complete obstruction of the central portal vein or its branches by PVTT may lead to collateral circulation development in most patients. However, research on tumor prognostic factors following treatment is limited, especially with regard to imaging findings, such as hepatic portal vein collateral circulation [7]. Radiotherapy (RT) is an effective treatment for HCC patients with PVTT [8,9]. RT, either alone or in combination with other therapies, can downstage tumors, thereby creating surgical opportunities for more advanced patients [10,11]. Computed tomography (CT) and magnetic resonance imaging (MRI) are currently commonly used to evaluate the treatment response by utilizing Response Evaluation Criteria in Solid Tumors (RECIST) version 1.1 [12], which is also employed in this work. This study was designed to identify imaging features of patients with HCC with PVTT who exhibited longer survival after combination therapy. We aimed to assess the laboratory examinations, pathophysiological indicators, and imaging features, as well as the relationship between the collateral circulation before and after the combined treatment strategy.

## 2. Materials and Methods

This retrospective study evaluated 26 patients with advanced-stage HCC and PVTT who survived for more than 1 year. These patients were selected from among 3884 HCC patients at Chang Gung Memorial Hospital, Chiayi Branch, between 1 January 2010 and 31 July 2021. We excluded those with a recorded survival of less than 1 year, those with concurrent cancers, those who received immunotherapy, those who had extrahepatic metastasis, and those who had an undefined tumor stage. Data were collected from the patients involved in this study, which included a detailed description of the overall clinical characteristics and the imaging features considered in the analysis.

Among the included patients, the initial tumor stage of 14 individuals was classified as T1,2, and 15 patients were classified as BCLC 0, A, B. These patients did not initially have tumor thrombosis; it only developed after failure of treatments such as surgery, radioablation, or transarterial chemoembolization. Additionally, 24 patients displayed liver functions that were classified as Child–Pugh A. Only the liver functions of two patients were classified as Child–Pugh B.

Systemic therapy (tyrosine kinase inhibitors, TKIs) was initiated following PVTT confirmation via imaging, with radiotherapy beginning 1 month later; we did not discontinue TKI use during the RT period unless adverse reactions arose. During the study period, sorafenib (400 mg or 200 mg twice daily) or lenvatinib (8–12 mg once daily) was used as a first-line drug based on the patient’s medication compliance. Eight patients switched to the second-line drug regorafenib due to disease progression (three cases) or adverse reactions (five cases), such as hand–foot syndrome, diarrhea, fatigue, rash, or desquamation.

For patients undergoing RT (VMAT), treatment was delivered using Synergy (Elekta LTD, Stockholm, Sweden) or EDGE (Varian Medical Systems, Palo Alto, CA, USA) Linacs. Patients undergoing RT fasted for at least three hours before simulation and each treatment session. A four-dimensional computed tomography (4D CT) scan was performed with abdominal compression to reduce respiratory motion. We did not administer medicine before or during RT. Most patients experienced abdominal pain after treatment but recovered on their own within weeks without medication. All participants finished RT without any signs of radiation-induced liver disease, such as jaundice, ascites, or elevated liver enzymes. The planned radiation dose, as determined by the radiation oncologist, was based on average intensity projection (AIP)-reconstructed images. RT doses were tailored to optimize PTV coverage, with 3000–5000 cGy over 10–20 fractions while maintaining dose constraints for organs at risk. The treatment response was assessed via imaging six months after RT.

**Figure 1:**
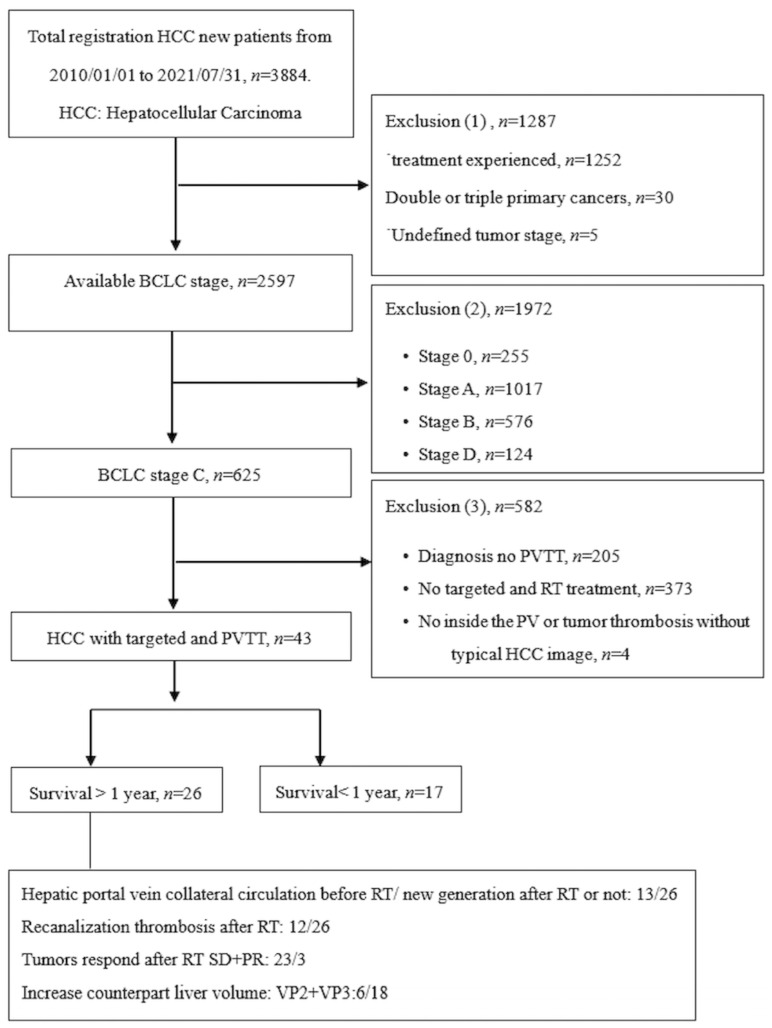
Patient enrolment flowchart.

An enrollment flowchart is shown in Figure 1; it provides a comprehensive description of the enrollment criteria and clearly illustrates the patient selection process. This study received approval from the Institutional Review Board of Chang Gung Memorial Hospital (IRB No: 202200399BO) on 31 March 2022.

Cross-sectional images obtained before treatment and 6 months after radiotherapy were reviewed using the Picture Archiving and Communication System (PACS). Two board-certified radiologists specializing in oncologic liver imaging (SS.H and WM.L) independently analyzed all CT and MRI examinations and reached consensus on every finding. The tumor response was assessed according to RECIST version 1.1, and portal vein tumor thrombus (PVTT) was retrospectively classified based on the Liver Cancer Study Group of Japan system. All imaging studies provided complete liver coverage with a reconstructed transverse slice thickness of 5 mm. Liver volume was measured using a manual tracing technique; the liver parenchyma was segmented by manually outlining the liver boundaries on every individual slice. Maximum craniocaudal diameters were assessed across all axial and coronal reconstructions using a dedicated PACS viewer (IMPAX EE R20, Agfa Healthcare, Mortsel, Belgium). Portal vein recanalization was defined as the reappearance of contrast medium within the portal vein lumen, occupying > 50% of the vessel diameter during the delayed phase post-injection. Collateral circulation—specifically cavernous transformation—was identified by the presence of multiple small, tortuous, enhancing vascular channels during the portal venous phase that replaced the expected single-lumen portal vein anatomy.

Continuous variables were expressed as mean ± standard deviation. Given the non-normal distribution of the data, the Wilcoxon signed-rank test was employed to compare paired pre- and post-intervention measurements. Categorical variables were analyzed using the McNemar test.

All statistical analyses were performed using SPSS software version 22.0 (SPSS Inc., Chicago, IL, USA). For all comparisons, a *p*-value < 0.05 was considered to indicate statistical significance.

## 3. Results

Table 1 summarizes patient demographics. Among the 26 patients with HCC, 18 were male (69.2%), and 8 were female (30.8%). Regarding viral etiology, 7 patients had hepatitis B virus (HBV), 13 had hepatitis C virus (HCV), and 4 had dual HBV/HCV infection. Liver cirrhosis was the most prevalent comorbidity (25 patients, 96.2%), followed by alcohol use (11 patients, 42.3%), hypertension (9 patients, 34.6%), and diabetes mellitus (4 patients, 15.4%).

Table 2 presents the laboratory and imaging findings obtained after radiotherapy. Serum AFP levels declined in 16 patients, with significantly greater improvement among those with a baseline AFP < 100 ng/mL (*p* = 0.022). Significant post-treatment changes were also observed in ALBI grade (*p* = 0.045) and prothrombin time (*p* = 0.008).

**Figure 2:**
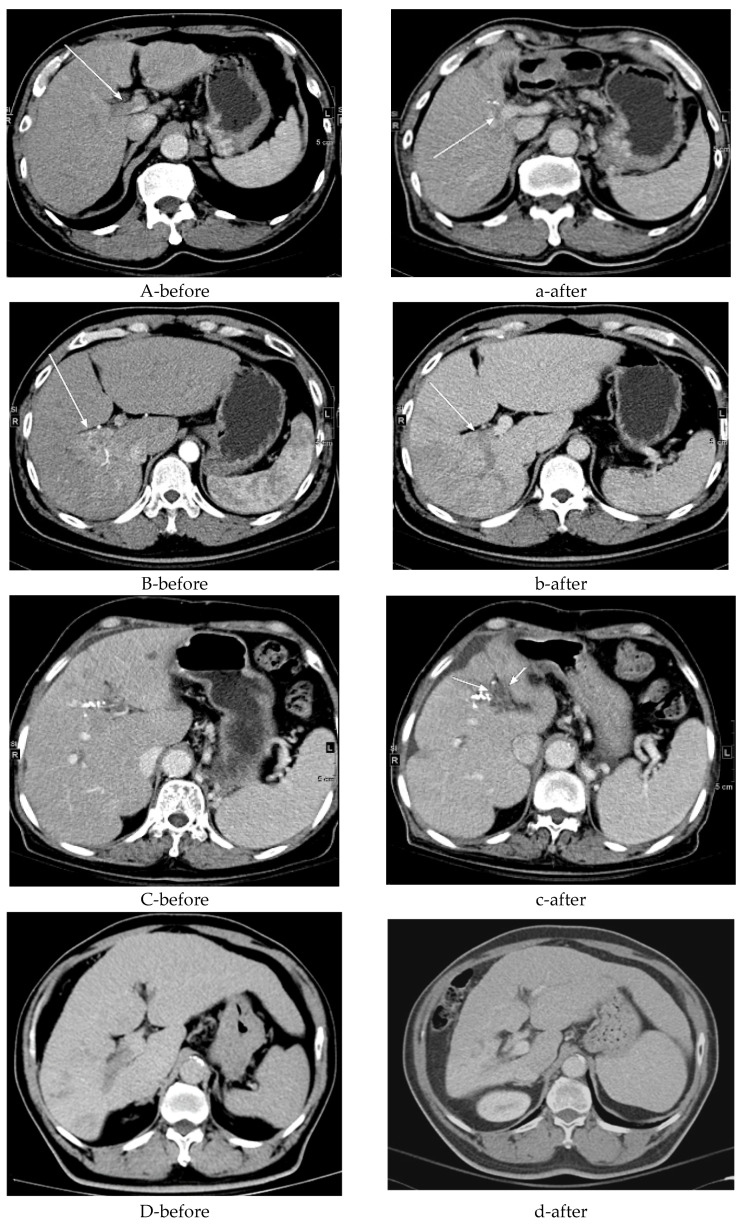

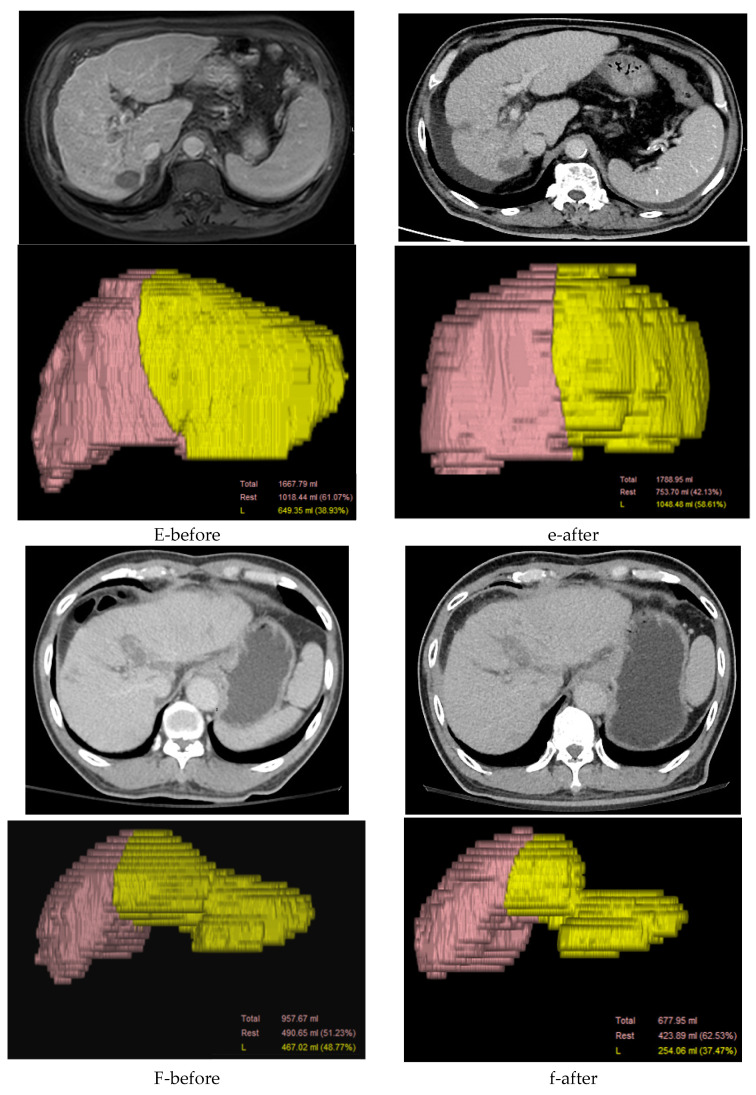
Detailed progression on computed tomography imaging. (**A**) A 70-year-old male with hepatitis B virus (HBV) and bilateral portal vein (PV) tumor thrombosis (see the white arrow) (**a**) exhibited partial recanalization of the right portal vein (PV) after receiving radiotherapy (RT) at 3000 cGy over 15 fractions (see the white arrow). (**B**) A 45-year-old male with hepatitis C virus (HCV) and right PV tumor thrombosis (see the white arrow) (**b**) did not exhibit recanalization of the right PV after receiving RT at 5000 cGy over 10 fractions (see the white arrow). (**C**) A 72-year-old female with HCV and left PV tumor thrombosis (**c**) exhibited new vessel formation around the PV after receiving RT at 3000 cGy over 15 fractions (see the white arrow). (**D**) A 58-year-old male with HBV and right PV tumor thrombosis showed (**d**) no new vessel formation after receiving RT at 5000 cGy over 20 fractions. (**E**-before) A 79-year-old male, a non-B and non-C patient (NBNC) with right VP3 (first-order portal vein invasion) tumor thrombosis, showed (**e**-after) a 61% increase in the counterpart liver volume post-RT. (**F**-before) A 72-year-old male HCV patient with left VP3 tumor thrombosis showed (**f**-after) a 13% decrease in the counterpart liver volume post-RT.

Twelve patients demonstrated recanalization of the portal vein tumor thrombus after external-beam radiotherapy—four with complete and eight with partial recanalization—based on 6-month follow-up imaging. Fourteen patients continued to show persistent occlusion despite treatment. Collateral circulation was identified in 7 patients before radiotherapy and in 6 afterward, whereas 13 patients never exhibited collateral flow. Intraparenchymal portal vein occlusion (VP2–VP3) was observed in 18 patients. Additionally, compensatory hypertrophy of the contralateral liver segment was noted in 6 patients; 12 patients showed no such change. Figure 2 illustrates these imaging findings.

## 4. Discussion

PVTT is an independent prognostic factor for unfavorable outcomes and reduces survival time. The abnormal expression of biomacromolecules (e.g., circular and long noncoding RNAs, stress-inducible protein 1, and PD-L1) in patients with HCC is strongly correlated with macrovascular invasion [13]. The recent advances in systemic and immune therapies for HCC have led to a wide array of medications, used in either single-agent or combination therapies [14]. Combination therapies involving locoregional treatments and systemic agents are increasingly favored for enhancing patient management and extending survival despite the complexities associated with PVTT in HCC. Several studies have identified the key prognostic factors influencing the management of HCC with PVTT. For example, the presence of portal vein invasion, AFP levels, and favorable tumor characteristics are critical for predicting patient survival outcomes [15,16]. Bone mineral density has also emerged as an independent survival predictor for patients with HCC and PVTT [17].

Local treatments such as RT improve tumor control and quality of life by alleviating symptoms in patients with HCC and vascular invasion or extrahepatic disease [18]. A multicenter study conducted in Korea by Im et al. [19] reported an improved median overall survival for patients with HCC and major portal vein invasion when using combination therapy (i.e., RT plus other modalities) versus RT alone (10.4 vs. 8.7 months, *p* = 0.023). Our study results demonstrate that in patients treated with systemic therapy (tyrosine kinase inhibitors) in combination with radiotherapy, recanalization of the tumor thrombus, collateral circulation, and increased liver volume were observed among long-term survivors.

Assessing the efficacy of RT for treating PVTT is challenging [20]. A previous study indicated that 70% of patients with PVTT showed a partial response within the first few weeks of RT, with most patients experiencing portal vein flow restoration [21]. In our study, thrombosis recanalization was observed in 12 patients (46.2%) (Figure 2A). Additionally, a thrombus with a maximum diameter ≤ 3 cm is a favorable factor for local response, serving as a potential imaging marker for effective PVTT control [22]. We also found that this phenomenon about restoration of portal vein blood flow was frequently noted in long-term survivors.

Collateral veins (e.g., para-choledochal veins) can facilitate nutrient and oxygen supply to hepatocytes via the portal vein, thereby potentially improving overall survival. In our study, the hepatic portal vein collateral circulation was established either before or after RT in 13 patients (50%) (Figure 2C). Hepatic fibrosis and cirrhosis caused by persistent liver injury are closely related to the liver reserve function and regeneration of the residual liver tissue. Intraparenchymal portal vein occlusion (VP2,3) stimulates the hypertrophy of the counterpart liver parenchyma with atrophy of the tumor part [23]. In our study, we observed an increase in the counterpart liver volume after RT in six patients (33.3%) (Figure 2E). Prognosis in these patients is challenging because of the highly heterogeneous tumor extent and characteristics. These imaging features appeared more frequently in long-term survivors, though their prognostic value has not been established and warrants further investigation. Overall, 23 patients (88.5%) exhibited either stable disease or a partial response based on RECIST 1.1, consequently contributing to increased overall survival.

The Child–Pugh score and ALBI grade are crucial assessments of liver function that directly influence patient survival and mortality [24]. In our cohort, the initial liver function was classified as Child–Pugh A in 24 patients (92.3%) and as ALBI grade I in 14 patients (53.8%). Following treatment, liver function improved or was maintained in 20 patients (76.9%) according to the Child–Pugh score and in 17 patients (65.4%) according to the ALBI grade. Concurrently, platelet count significantly dropped from 96.5 to 63 × 103/Ul (*p* < 0.001) in long-term survivors after treatment. This observation was particularly relevant because thrombocytopenia has been linked to a prolonged recurrence-free survival and overall survival post-hepatectomy [25]. Furthermore, serum AFP levels improved in 16 patients, with a remarkable decrease observed in those with an initial AFP below 100 ng/mL Tumor control, preserved liver function, and reduced platelet count were commonly observed in this cohort.

Adverse effects may occur with the combined treatment strategies; thus, their safety profiles remain an important focal point. While combination therapies show improved survival rates, they also carry risks of increased adverse events. According to previous studies, the incidence of severe adverse events can be effectively managed, making combination therapies a viable option for patients [26,27]. The management of HCC with portal vein tumor thrombosis presents a unique set of challenges that necessitate innovative treatment strategies leveraging local and systemic therapies.

This study has limitations: First, the retrospective, single-center design and small sample size limit the generalizability of the findings. The cohort was restricted to long-term survivors, introducing an inherent selection bias. Without a comparative control group of short-term survivors, these findings must be interpreted as descriptive characteristics rather than definitive prognostic factors or direct evidence of treatment efficacy. Second, collecting clinical data from medical records was difficult and time-consuming. The treatment course of the 26 patients involved in this study was complicated, and extending it further would be difficult. A detailed study with an adequate sample size would enhance the value of the analysis.

Nevertheless, our data was still statistically significant despite the small sample size. Third, the time period of patient enrollment was extended, with the RT group being enrolled later. This led to heterogeneity among the patients included in this study, especially considering the rapid advancements in immuno- and targeted systemic therapy. Larger multicenter studies are required to validate our results.

## 5. Conclusions

In HCC patients with PVTT who survived longer than one year after combined therapy, favorable imaging characteristics include recanalization of the tumor thrombus, formation of collateral circulation, and compensatory liver hypertrophy. Preserved baseline liver function remains a critical feature of this high-risk subgroup. Future prospective studies with larger, comparative cohorts are necessary to validate these observations.

## Figures and Tables

**Table 1:** Basic characteristics of the overall study cohort.

Variables	Survival > 1 Year (*n* = 26)
Age, years (mean ± SD)	61.9 ± 9.1
Gender	
Male, *n* (%)	18 (69.2)
Female, *n* (%)	8 (30.8)
Hepatitis	
NBNC, *n* (%)	2 (7.7)
HBV, *n* (%)	7 (26.9)
HCV, *n* (%)	13 (50.0)
HBV + HCV, *n* (%)	4 (15.4)
Initial HCC American Joint Committee on Cancer (AJCC) stage	
T1, *n* (%)	8 (30.8)
T2, *n* (%)	6 (23.1)
T3, *n* (%)	9 (34.6)
T4, *n* (%)	3 (11.5)
Initial Barcelona Clinic Liver Cancer (BCLC) stage	
0 + A + B, *n* (%)	15 (57.7)
C, *n* (%)	11 (42.3)
Child–Pugh grade	
A	24 (92.3)
B	2 (7.7)
C	-
Albumin–bilirubin (ALBI) grade	
I	14 (53.8)
II	11 (42.3)
III	1 (3.8)
PVTT type	
VP1 + VP2, *n* (%)	7 (26.9)
VP3 + VP4, *n* (%)	19 (73.1)
Cirrhosis, *n* (%)	25 (96.2)
Diabetes mellitus, *n* (%)	4 (15.4)
Hypertension, *n* (%)	9 (34.6)
Alcohol, *n* (%)	11 (42.3)

NBNC: non-B, non-C; HBV: hepatitis B virus; HCV: hepatitis C virus; PVTT: portal vein tumor thrombosis.

**Table 2:** Laboratory and imaging indicators obtained after radiotherapy in 26 patients who survived for more than 1 year.

Variables	Before RT (*n* = 26)	After RT (*n* = 26)	*p*-Value	Improved	Maintained	Worsened
AFP, ng/mL (median; IQR)	13.4 (6.9–136.8)	8.4 (4.2–21.4)	0.024 *	16	3	7
<100 ng/mL, *n* (%)	18 (69.2)	23 (88.5)	0.022 *			
≥100 ng/mL, *n* (%)	8 (30.8)	3 (11.5)				
Child–Pugh grade			0.075	0	20	6
A, *n* (%)	24 (92.3)	18 (69.2)				
B, *n* (%)	2 (7.7)	8 (30.8)				
Albumin–bilirubin (ALBI) grade			0.045 *	1	16	9
I	14 (53.8)	6 (23.1)				
II + III	12 (46.2)	20 (76.9)				
AST (U/L; median, IQR)	62.5 (51.8–73.5)	67 (62–77.5)	0.226	5	7	14
ALT (U/L; median, IQR)	49.5 (31.8–66.5)	49 (34–63)	0.459	6	10	10
Platelet count, 10^3^/Ul (median, IQR)	96.5 (62.5–125.5)	63 (44.2–78.8)	<0.001 *	2	5	19
Prothrombin time, s (median, IQR)	11.2 (10.5–12.4)	11.4 (10.5–12)	0.396	0	24	2
Recanalization thrombosis after RT		12				
Formation of hepatic portal vein collateral circulation	7	6				
VP2 + VP3 counterpart liver volume increase after RT						
Yes		6				
No		12				
PVTT belongs to VP4		8				
Tumors respond after RT						
SD + PR		23				
PD		3				

IQR, interquartile range; AFP, alpha-fetoprotein; AST, aspartate aminotransferase; ALT, alanine transaminase; RT, radiotherapy; SD, stable disease; PR, partial response; PD, progressive disease; * *p* < 0.05.

## Data Availability

All data analyzed are included in this published article. The original data are available upon reasonable request from the corresponding author.
